# Case report: Identification of potential prognosis-related LAG3 overexpression and DICER1 mutation in pituitary carcinoma: two cases

**DOI:** 10.3389/fnins.2023.1191596

**Published:** 2023-10-12

**Authors:** Yi Zhang, Victoria Li, Jifang Liu, Huijuan Zhu, Lin Lu, Hui Pan, Renzhi Wang, Kan Deng, Yong Yao

**Affiliations:** ^1^Department of Neurosurgery, Peking Union Medical College Hospital, Chinese Academy of Medical Science and Peking Union Medical College, Beijing, China; ^2^Faculty of Medicine, Dentistry and Health Sciences, The University of Melbourne, Melbourne, VIC, Australia; ^3^Department of Endocrinology, Peking Union Medical College Hospital, Chinese Academy of Medical Science and Peking Union Medical College, Beijing, China

**Keywords:** pituitary carcinoma, DICER1, LAG3, metastatic PitNET, prognosis

## Abstract

Metastatic PitNETs are a rare life-threatening condition with poor prognosis and documentation. Due to the scarce literature and lack of precise treatment, we hope to better characterise PitNET using the next-generation whole exon sequencing (WES) and RNA sequencing. This case study outlines a 54 years-old man and a 52 years-old woman who were both diagnosed with PitNET and analysis of peripheral blood and tumours were performed by WES and RNA sequencing. Analysis showed that DICER1 mutations in precancerous lesions and LAG3 overexpression were significant in aiding the prognosis and diagnosis of PitNETs. The first case with overexpressed LAG3 and DICER1 mutation died 26 months later, and the second case with LAG3 overexpression achieved partial remission. This study revealed that heightened expression of LAG3 offered promising targets for ICI and mutations in DICER1 could provide markers for effective diagnosis and prognosis.

## Introduction

Pituitary carcinoma or metastatic Pituitary Neuroendocrine Tumours (PitNET) are an extremely rare clinical entity accounting for 0.1% of diagnosed intracranial tumours with less than 200 cases documented globally to date (Daly et al., 2009). However, 75% of these cases were diagnosed via autopsy (Cusimano et al., 1994), strongly suggesting difficulty in prognosis as many cases are largely asymptomatic or clinically silent (Cartwright et al., 1994; Pernicone et al., 1997). PitNETs most commonly presents itself in patients around 30–50 with no preference in gender (Kaltsas and Grossman, 1998) and is a life-threatening condition with an average survival after metastatic diagnosis of 4 years (Mountcastle et al., 1989; Popovic et al., 1991; Ragel and Couldwell, 2004; Xu et al., 2020). The diagnosis of PitNET currently relies on histopathological results including a Ki-67 cell proliferation index of greater than 20%, MRI imaging, and clinical history (Ntyonga-Pono et al., 1999). Due to a scarcity in literature and lack of precise prognosis, improving the understanding of genetic markers is necessary. Here, we present two cases of PitNET undergoing genomic and transcriptomic analysis of tumour mass and peripheral blood by WES and RNA sequencing (Chen et al., 2021; Zhang et al., 2022; Zhou et al., 2022). It was found that the overexpression of LAG3 and somatic mutations in DICER1 may be accountable for tumorigenesis and metastasis. LAG3 has been identified as an inhibitor that limits the expansion and memory pool of CD8+ T cells, and found to play a role in colorectal, oesophageal, and pituitary tumorigenesis among others (Andrews et al., 2022). Germline DICER1 mutations can increase the risk of tumorigenesis, and the second hit disrupts DICER1 regulated apoptosis, protein translational control, and microRNA processing (de Kock et al., 2014; Robertson et al., 2018).

## Case presentation

### Case 1

A 54 years-old male presenting with a cortical adenoma which was previously suspected to be Cushing’s disease recurrence had received insulin intravenous infusion and nifedipine sustained-release tablets following a frontotemporal craniotomy. Displaying little improvement, the patient received radiotherapy (5Gy/fraction) in the sellar area.

**Figure 1:**
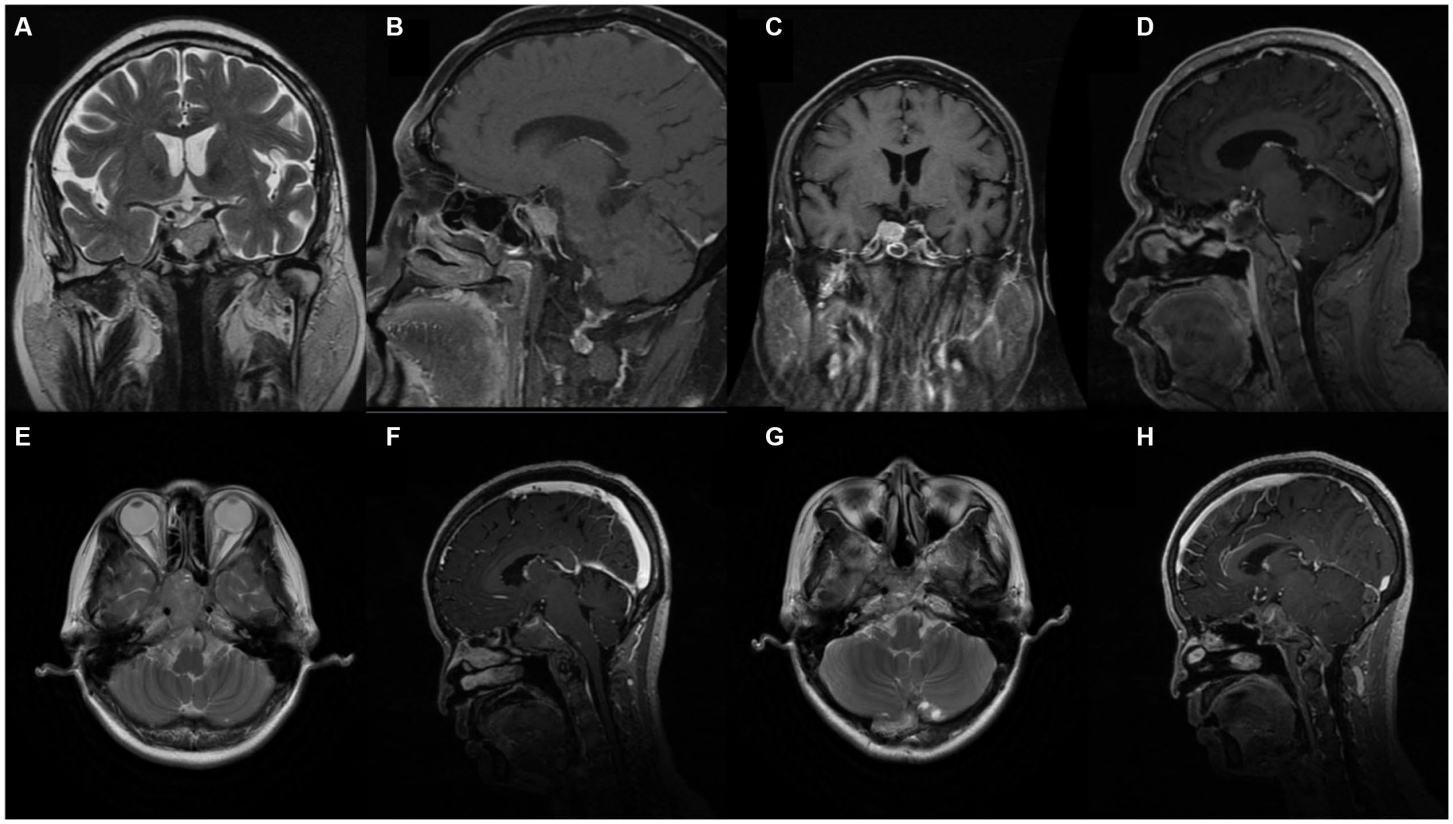
Pre-operative enhanced Magnetic Resonance Imaging (MRI) revealed large mass lesion in the sellar region. (**A, B**) Case 1. Pre-operative MRI demonstrated large tumour in the pituitary gland involving left sinus. (**C, D**) Case 1. Post-operative enhanced MRI showing decreases tumour mass and cancer metastasis. (**E, F**) Case 2. Pre-operative MRI with heterogeneous enhancement. (**G, H**) Case 2. Post-operative MRI.

Upon admission to our institution, enhanced MRI revealed a large tumor in the pituitary gland (3.95 cm × 1.69 cm × 1.61 cm) and the sellar region was enlarged (Figures 1A,B). The lesion showed enhancement homogenously and the optic junction was not observed. The possibility of a pituitary macroadenoma involving the left cavernous sinus was considered.

Surgical removal of the tumor was performed through a neuroendoscopic resection in the sellar area of the nasal sphenoid sinus in May 2020. Postoperative pathology revealed a large pituitary adenoma showing active growth (Ki-67 10%). Immunohistochemical results were positive for AE1/AE3, CAM5.2 and T-PIT and negative for LH, PIT-1, ER, GH, P53, PRL, TSH, and FSH. ACTH was checked 1 day after and was 34.1 pg./mL and F 24.8ug/dL. Five days after the operation, ACTH was 21.2 pg./mL and F 14.5ug/dL, respectively. A comparison of pituitary MRI pre and post operation found a slight reduction in the residual tumor on the right wing (1.2 cm × 1.4 cm). The enhanced MRI (Figures 1C,D) also showed scattered abnormal signals in the operative regions. Metastasis was considered in the frontal lobe.

**Figure 2:**
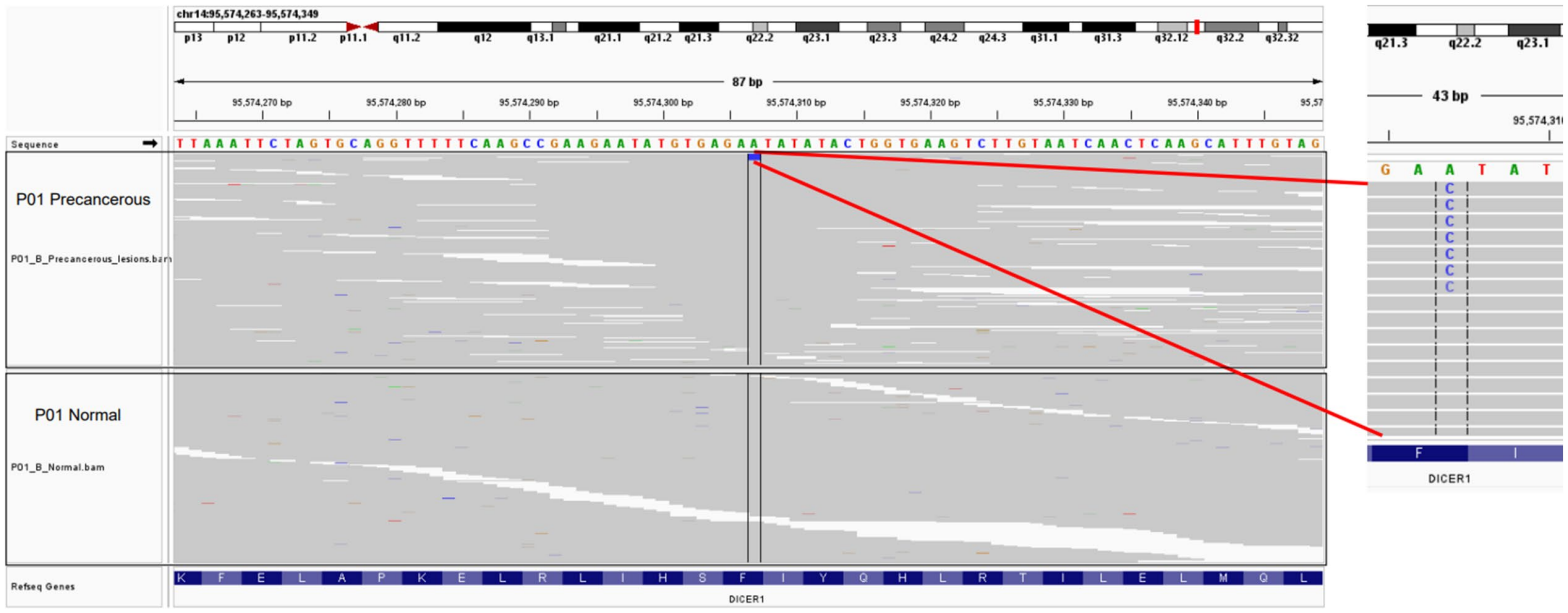
Visualization of selected point mutation of the DICER1 gene in precancerous lesions with the IGV tool. The upper panel represents the precancerous tissue of P01, while the lower panel represents the matched control samples.

**Figure 3:**
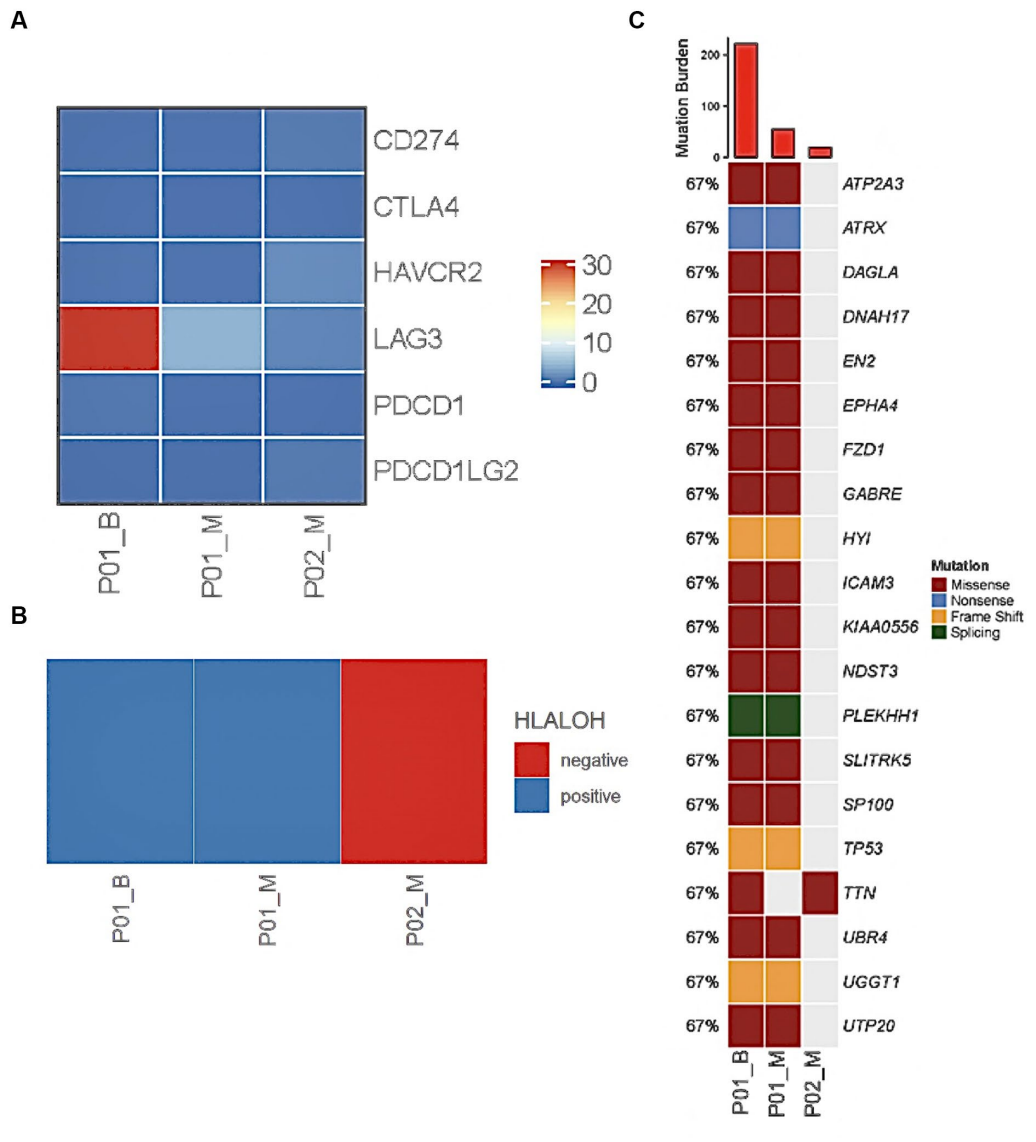
WES and RNA sequencing reveal genetic abnormality in cerebrospinal fluid and tumour of PC patients. LAG3 was overexpressed in Case 1 benign sample and showed slight heightened expression in malignant tumours of Case 1 and 2 **(A)**. HLALOH staining was positive in Case 1 **(B)**. Tumour mutation burden was high in tumour samples at the DICER1 gene **(C)**.

The patient was administered oral temozolomide and gamma knife treatment. Because of weight gain and decreased eye acuity, the patient was then readmitted to our unit in December of 2020 under the diagnosis of aggressive PitNET and a left frontal lobe lesion biopsy was performed. Cerebrospinal cytology and genomic analysis revealed significant and differential genes. The DICER1 gene p.Phe854Val was only mutated in precancerous lesions, implying that ACTH regulating miRNA may be abnormal. Direct comparison of base sequences at the same gene localization of matched peripheral blood mononuclear cells (PBMC) showed no variant verifying the change in the benign tumor genome as a somatic mutation (Figure 2). HLA LOH occurred in both the benign and malignant tumors. LAG3 was found to be highly expressed in hyperactive T cells, especially in ACTH secreting pituitary adenomas along with copy number amplifications of genes including PTPN11, PDGFRB, FGFR1, KDM5A, CCNE1, and CEBPA (Figures 3A,B). Suppressed T cells were in large proportion compared to ACTH enhanced T cells suggesting an immunosuppressive microenvironment caused by LAG3. Comparing the patient’s benign and malignant tumors, a significant gain was found in many oncogenes (Figure 3C). Notably, the microsatellites of non-coding regions were largely unstable. DNA mismatch repair signatures reflected the possible mutational processes. Upon discharge, the patient reported no physical discomfort or abnormalities. The patient died 2 years later due to circulatory respiratory failure.

### Case 2

A 52 years-old woman with history of an untreated pituitary tumour underwent pituitary adenoma resection in February 2015 and July 2018 before being admitted to our hospital in December 2020. A pre-operative MRI scan (Figures 1E,F) with enhancement indicated multiple abnormal diffuse signals with obvious enhancement in the sub-tentorial cerebellum and the foramen magnum. An excision and biopsy of the posterior occipital lobe was performed under consideration of PitNET. Post-operative immunohistochemical results showed positive ACTH, T-PIT, AE1/AE3, CAM5.2, and CgA. LH, GH, P53, PRL, TSH, FSH, and PIT-1. Ki-67 cell proliferation index was 5%. ECG monitoring, oxygen, fluid replacement, pain relief, anti-epileptic, anti-infection, and potassium supplementation were administered to the patient following surgery until discharge. An MRI performed following the surgery showed irregular soft tissue in the sellar and parasellar regions (Figures 1G,H).

Genetic analysis indicated that the patient did not exhibit HLA LOH (Figure 3B), however a mutation of TTN was found (Figure 3C). Genomic analysis revealed that tumor mutation burden was high and LAG3 was overexpressed in ACTH functioning tumors in comparison to metastatic lesions (Figure 3A: ACTH vs. PC TMB: 222 Mut/Mb vs. 19 Mut/Mb). The patient is in partial remission after chemotherapy, radiotherapy gamma knife treatment and oral temozolomide.

## Discussion

Pituitary tumours mostly remain benign and display a slow rate of growth (Melmed, 2003), therefore metastasis is very rare. Currently, it is difficult to diagnose PitNET due to its varying clinical presentations. PitNETs can present asymptomatically, as mass lesions, or as functional endocrine neoplasms which are the most common (Heaney, 2011). Cushing’s disease is commonly observed in patients with ACTH secreting tumours as observed in Case 1 (Ragel and Couldwell, 2004; Vandeva et al., 2019). Other syndromes that have been found to predispose PitNET include Multiple Endocrine Neoplasia (MEN) (Asa et al., 2022) which has been linked to tumorigenesis of the anterior pituitary, as well as McCune-Albright syndrome, Carney complex, and less frequently, succinate dehydrogenase and MYC-associated factor X (MAX) related syndromes (Barry and Korbonits, 2020). Literature is limited in the genetic understanding of PitNET, however germline mutations such as aryl hydrocarbon receptor-interacting proteins (AIP), CDH23, and DICER have been found to predispose pituitary adenoma (Zhang et al., 2017; Lim and Korbonits, 2018; Nosé et al., 2022) and mutations in ATRX, TP53, NF1, and PTEN could contribute to carcinoma (Casar-Borota et al., 2020; Sumisławski et al., 2022). There is no optimal treatment for PitNET, however surgery and radiation has been the most widely adopted treatment. Surgery primarily serves to relieve compressive symptoms and prevent excessive hormone secretion however is not sufficient in preventing metastasis (Lehman et al., 2003). Hence, radiotherapy is conducted in hopes of achieving local control. Additionally, literature suggests that the administration of temozolomide is effective in increasing survival in PitNET patients (McCormack, 2022). An increasing need for differential genes in aiding both the prognosis and effective treatment of PitNET is evident. Hence, we present two more differential genes, DICER1 and LAG3, which pose significance in prognosis and treatment.

LAG3 which is mainly expressed on activated T cells acts as a receptor protein (Darvin et al., 2018) and has been identified as a promising target for immunotherapy in cancer in several studies (Villadolid and Amin, 2015; Feola et al., 2022). It has been found to enhance tumour growth by inhibiting immune microenvironments (Shi et al., 2022). The regulation of LAG3 is tightly associated with solid tumour immune infiltration, however the prognostic reliability of these checkpoints remains controversial. Recently, there has been burgeoning research in this novel field for combining LAG3 and PD-1 as therapeutic targets (Tu et al., 2020). Ascierto et al. (2017) found that monoclonal antibodies targeted towards LAG3 effectively treated melanoma populations. Furthermore, immunotherapy utilising immune checkpoint inhibitors (ICIs) has proven to be effective in almost 50% of cases targeting PDL1 in PitNET (Feola et al., 2022). Here, we offer LAG3 as another novel ICI target for PitNETs. Currently, animal models have also provided evidence for successful PitNET gene therapy utilising viral vectors to deliver genes with minimal toxicity and long-term transgene expression (Huo et al., 2022b). DICER1 mutations have also been documented in numerous studies of both benign and malignant tumours, including in the pituitary gland and recently has been found to affect miRNA and gene regulation (Seilicovich et al., 2005; Robertson et al., 2018). Liu et al. (2021) found in a clinical study of 17 teenagers, that 16 were found to have blastomas attributed to DICER1 abnormalities. This affirms the potential in screening for DICER1 mutations in patients suspected of PitNET and has applications in earlier prognosis which is necessary.

Further understanding of prognostic markers like DICER1 and gene therapy targets such as LAG3 are imperative in aiding earlier diagnosis and more effective treatments for PitNET. In this study however, one potential limitation was the lack of functional experiments. Functional studies to validate whether the molecular changes differentiate with other potential passenger mutations or a VUS, and how the mutation affects the pathogenesis are warranted.

## Conclusion

We have evaluated two rare cases of PitNET and presented two differential genes with extremely promising prognosis via DICER1 screening as well as potential ICI and gene therapy treatments targeting LAG3. There are significant applications for developing more precise and accurate diagnoses as well as more effective treatment for PitNET, however further studies in the application of gene therapy should be performed to validate such findings.

## Data availability statement

The datasets presented in this study can be found in online repositories. The names of the repository/repositories and accession number(s) can be found in the article/supplementary material.

## Ethics statement

The studies involving human participants were reviewed and approved by PUMCH Institutional Review Board. The patients/participants provided their written informed consent to participate in this study. Written informed consent was obtained from the individual(s) for the publication of any potentially identifiable images or data included in this article.

## Author contributions

YY, RW, and YZ: conception and design. HP and YZ: development of methodology. YZ and KD: acquisition and analysis of data. YZ, VL, and JL: writing, review, and revision of manuscript. HZ and LL: technical and material support. YY, RW, and HZ: study supervision. All authors contributed to the article and approved the submitted version.

## References

[ref1] Andrews L. C. Cillo A. R. Karapetyan L. Kirkwood J. M. Workman C. J. Vignali D. A. A. (2022). Molecular pathways and mechanisms of LAG3 in cancer therapy. Clin. Cancer Res. 28, 5030–5039. doi: 10.1158/1078-0432.ccr-21-2390, PMID: 35579997 PMC9669281

[ref2] Asa S. L. Mete O. Perry A. Osamura R. Y. (2022). Overview of the 2022 WHO classification of pituitary tumors. Endocr. Pathol. 33, 6–26. doi: 10.1007/s12022-022-09703-7, PMID: 35291028

[ref3] Ascierto P. Bono P. Bhatia S. Melero I. Nyakas M. Svane I. M.. (2017). Efficacy of BMS-986016, a monoclonal antibody that targets lymphocyte activation gene-3 (LAG-3), in combination with nivolumab in pts with melanoma who progressed during prior anti–PD-1/PD-L1 therapy (mel prior IO) in all-comer and biomarker-enriched populations. Ann. Oncol. 28, v611–v612. doi: 10.1093/annonc/mdx440.011

[ref4] Barry S. Korbonits M. (2020). Update on the genetics of pituitary tumors. Endocrinol. Metab. Clin. N. Am. 49, 433–452. doi: 10.1016/j.ecl.2020.05.005 32741481

[ref5] Cartwright D. M. Miller T. R. Nasr A. J. (1994). Fine-needle aspiration biopsy of pituitary carcinoma with cervical lymph node metastases: a report of two cases and review of the literature. Diagn. Cytopathol. 11, 68–73. doi: 10.1002/dc.2840110116, PMID: 7956665

[ref6] Casar-Borota O. Boldt H. B. Engström B. E. Andersen M. Baussart B. Bengtsson D.. (2020). Corticotroph aggressive pituitary tumors and carcinomas frequently harbor ATRX mutations. J. Clin. Endocrinol. Metab. 106, e1183–e1194. doi: 10.1210/clinem/dgaa749, PMID: 33106857 PMC7993578

[ref7] Chen K. Bai J. Reuben J. M. Zhao H. Kang G. Zhang C.. (2021). Multiomics analysis reveals distinct immunogenomic features of lung cancer with ground-glass opacity. Am. J. Respir. Crit. Care Med. 204, 1180–1192. doi: 10.1164/rccm.202101-0119oc, PMID: 34473939 PMC8759311

[ref8] Cusimano M. D. Ohori P. Martinez A. J. Jungreis C. Wright D. C. (1994). Pituitary carcinoma. Skull Base Surg. 4, 46–51. doi: 10.1055/s-2008-1058989, PMID: 17170926 PMC1656463

[ref9] Daly A. F. Tichomirowa M. A. Beckers A. (2009). The epidemiology and genetics of pituitary adenomas. Best Pract. Res. Clin. Endocrinol. Metab. 23, 543–554. doi: 10.1016/j.beem.2009.05.008 19945022

[ref10] Darvin P. Toor S. M. Sasidharan N. V. Elkord E. (2018). Immune checkpoint inhibitors: recent progress and potential biomarkers. Exp. Mol. Med. 50, 1–11. doi: 10.1038/s12276-018-0191-1, PMID: 30546008 PMC6292890

[ref11] de Kock L. Sabbaghian N. Plourde F. Srivastava A. Weber E. W. Bouron-Dal Soglio D.. (2014). Pituitary blastoma: a pathognomonic feature of germ-line DICER1 mutations. Acta Neuropathol. 128, 111–122. doi: 10.1007/s00401-014-1285-z, PMID: 24839956 PMC4129448

[ref12] Feola T. Carbonara F. Verrico M. Di Crescenzo R. M. Gianno F. Colonnese C.. (2022). Immunotherapy for aggressive and metastatic pituitary neuroendocrine tumors (PitNETs): state-of-the art. Cancers 14:4093. doi: 10.3390/cancers14174093, PMID: 36077631 PMC9454884

[ref13] Heaney A. P. (2011). Clinical review: pituitary carcinoma: difficult diagnosis and treatment. J. Clin. Endocrinol. Metab. 96, 3649–3660. doi: 10.1210/jc.2011-2031, PMID: 21956419 PMC3277423

[ref14] Huo J. L. Wang Y. T. Fu W. J. Lu N. Liu Z. S. (2022b). The promising immune checkpoint LAG-3 in cancer immunotherapy: from basic research to clinical application. Front. Immunol. 13:956090. doi: 10.3389/fimmu.2022.956090, PMID: 35958563 PMC9361790

[ref15] Kaltsas G. A. Grossman A. B. (1998). Malignant pituitary tumours. Pituitary 1, 69–81. doi: 10.1023/a:1009975009924 11081185

[ref16] Lehman N. L. Horoupian D. S. Harsh G. R. (2003). Synchronous subarachnoid drop metastases from a pituitary adenoma with multiple recurrences. Case report. J. Neurosurg. 98, 1120–1123. doi: 10.3171/jns.2003.98.5.1120, PMID: 12744376

[ref17] Lim C. S. Korbonits M. (2018). Update on the CLINICOPATHOLOGY of pituitary adenomas. Endocr. Pract. 24, 473–488. doi: 10.4158/ep-2018-0034, PMID: 29498920

[ref18] Liu A. P. Y. Kelsey M. M. Sabbaghian N. Park S. H. Deal C. L. Esbenshade A. J.. (2021). Clinical outcomes and complications of pituitary Blastoma. J. Clin. Endocrinol. Metab. 106, 351–363. doi: 10.1210/clinem/dgaa857, PMID: 33236116 PMC7823240

[ref19] McCormack A. (2022). Temozolomide in aggressive pituitary tumours and pituitary carcinomas. Best Pract. Res. Clin. Endocrinol. Metab. 36:101713. doi: 10.1016/j.beem.2022.101713, PMID: 36274026

[ref20] Melmed S. (2003). Mechanisms for pituitary tumorigenesis: the plastic pituitary. J. Clin. Investig. 112, 1603–1618. doi: 10.1172/jci20401, PMID: 14660734 PMC281651

[ref21] Mountcastle R. B. Roof B. S. Mayfield R. K. Mordes D. B. Sagel J. Biggs P. J.. (1989). Pituitary adenocarcinoma in an acromegalic patient: response to bromocriptine and pituitary testing: a review of the literature on 36 cases of pituitary carcinoma. Am J Med Sci 298, 109–118. doi: 10.1097/00000441-198908000-00007, PMID: 2669475

[ref22] Nosé V. Gill A. J. Teijeiro J. M. B. Perren A. Erickson L. A. (2022). Overview of the 2022 WHO classification of familial endocrine tumor syndromes. Endocr. Pathol. 33, 197–227. doi: 10.1007/s12022-022-09705-5, PMID: 35285003

[ref23] Ntyonga-Pono M. P. Thomopoulos P. Luton J. P. (1999). Les métastases hypophysaires. 3 observations (Pituitary metastases. 3 cases). Presse Med. 28, 1567–1571. PMID: 10544705

[ref24] Pernicone P. J. Scheithauer B. W. Sebo T. J. Kovacs K. T. Horvath E. Young W. F.. (1997). Pituitary carcinoma: a clinicopathologic study of 15 cases. Cancer 79, 804–812. doi: 10.1002/(sici)1097-0142(19970215)79:4<804::aid-cncr18>3.0.co;2-3 9024719

[ref25] Popovic E. A. Vattuone J. R. Siu K. H. Busmanis I. Pullar M. J. Dowling J. (1991). Malignant prolactinomas. Neurosurgery 29, 127–130. doi: 10.1097/00006123-199107000-00023 1870674

[ref26] Ragel B. T. Couldwell W. T. (2004). Pituitary carcinoma: a review of the literature. Neurosurg. Focus. 16, 1–9. doi: 10.3171/foc.2004.16.4.8 15191336

[ref27] Robertson J. Jorcyk C. L. Oxford J. T. (2018). DICER1 syndrome: DICER1 mutations in rare cancers. Cancers 10:143. doi: 10.3390/cancers10050143 29762508 PMC5977116

[ref28] Seilicovich A. Pisera D. Sciascia S. A. Candolfi M. Puntel M. Xiong W.. (2005). Gene therapy for pituitary tumors. Curr. Gene Ther. 5, 559–572. doi: 10.2174/156652305774964721, PMID: 16457646 PMC2696484

[ref29] Shi A. P. Tang X. Y. Xiong Y. L. Zheng K. F. Liu Y. J. Shi X. G.. (2022). Immune checkpoint LAG3 and its ligand FGL1 in cancer. Front. Immunol. 12:785091. doi: 10.3389/fimmu.2021.785091, PMID: 35111155 PMC8801495

[ref30] Sumisławski P. Rotermund R. Klose S. Lautenbach A. Wefers A. K. Soltwedel C.. (2022). ACTH-secreting pituitary carcinoma with TP53, NF1, ATRX, and PTEN mutations case report and review of the literature. Endocrine 76, 228–236. doi: 10.1007/s12020-021-02954-0, PMID: 35171439 PMC8986667

[ref31] Tu L. Guan R. Yang H. Zhou Y. Hong W. Ma L.. (2020). Assessment of the expression of the immune checkpoint molecules PD-1, CTLA4, TIM-3, and LAG-3 across different cancers in relation to treatment response, tumor-infiltrating immune cells and survival. Int. J. Cancer 147, 423–439. doi: 10.1002/ijc.32785, PMID: 31721169

[ref32] Vandeva S. Daly A. Petrossians P. Zacharieva S. Beckers A. (2019). Somatic and germline mutations in the pathogenesis of pituitary adenomas. Eur. J. Endocrinol. 181, R235–R254. doi: 10.1530/eje-19-0602 31658440

[ref33] Villadolid J. Amin A. (2015). Immune checkpoint inhibitors in clinical practice: update on management of immune-related toxicities. Transl. Lung Cancer Res. 4, 560–575. doi: 10.3978/j.issn.2218-6751.2015.06.06, PMID: 26629425 PMC4630514

[ref34] Xu L. Khaddour K. Chen J. Rich K. M. Perrin R. J. Campian J. L. (2020). Pituitary carcinoma: two case reports and review of literature. World J. Clin. Oncol. 11, 91–102. doi: 10.5306/wjco.v11.i2.91, PMID: 32133278 PMC7046923

[ref35] Zhang Q. Peng C. Song J. Zhang Y. Chen J. Song Z.. (2017). Germline mutations in CDH23, encoding cadherin-related 23, are associated with both familial and sporadic pituitary adenomas. Am. J. Hum. Genet. 100, 817–823. doi: 10.1016/j.ajhg.2017.03.011, PMID: 28413019 PMC5420349

[ref36] Zhang S. Xiao X. Zhu X. Chen X. Zhang X. Xiang J.. (2022). Dysregulated immune and metabolic microenvironment is associated with the post-operative relapse in stage I non-small cell lung Cancer. Cancers 14:3061. doi: 10.3390/cancers14133061, PMID: 35804832 PMC9265031

[ref37] Zhou Z. Ding Z. Yuan J. Shen S. Jian H. Tan Q.. (2022). Homologous recombination deficiency (HRD) can predict the therapeutic outcomes of immuno-neoadjuvant therapy in NSCLC patients. J. Hematol. Oncol. 15:62. doi: 10.1186/s13045-022-01283-7, PMID: 35585646 PMC9118717

